# MEK inhibition appears to improve symptom control in primary *NRAS*-driven CNS melanoma in children

**DOI:** 10.1038/bjc.2017.49

**Published:** 2017-03-02

**Authors:** Veronica A Kinsler, Patricia O'Hare, Thomas Jacques, Darren Hargrave, Olga Slater

**Affiliations:** 1 Paediatric Dermatology, Great Ormond Street Hospital for Children NHS Foundation Trust, London WC1N 3JH, UK; 2 Genetics and Genomic Medicine, UCL Institute of Child Health, 30 Guilford Street, London WC1N 1EH, UK; 3 Paediatric Oncology, Great Ormond Street Hospital for Children NHS Foundation Trust, London WC1N 3JH, UK; 4 Paediatric Histopathology, Great Ormond Street Hospital for Children NHS Foundation Trust, London WC1N 3JH, UK; 5 Developmental Biology and Cancer Programme, UCL Institute of Child Health, 30 Guilford Street, London WC1N 1EH, UK

**Keywords:** melanoma, CNS, *NRAS*, trametinib, MEK inhibitor, paediatric, congenital melanocytic naevus

## Abstract

**Background:**

Primary melanoma of the CNS in children is extremely rare, and usually linked to congenital melanocytic naevus syndrome, caused by mosaicism for oncogenic *NRAS* mutations. Outcome is fatal in all cases. Data from murine and *in vitro* studies suggest that MEK inhibition is a possible therapeutic option.

**Methods:**

Four children with *NRAS*-mutated CNS melanoma were treated with Trametinib on a compassionate basis.

**Results:**

All four had an improvement in symptoms and objectively in signs. These varied from mild improvement for 1 month, to a sustained symptom-free period of 9 months in one case. In all cases there was eventual disease progression through treatment, followed by rapid death after discontinuation. There were no clinically-significant side effects.

**Conclusions:**

Trametinib is the first therapy to show any objective or measurable effect in *NRAS*-mutated primary CNS melanoma, with few side effects in this small series. The role of this therapy should be explored further in this rare paediatric tumour.

Primary CNS melanoma in childhood is extremely rare (CRUK, 2016), usually but not exclusively arising in the context of congenital melanocytic naevus (CMN) syndrome (unpublished data from our tertiary referral paediatric oncology department). CMN syndrome is the association of congenital moles with extra-cutaneous features such as congenital neurological malformations (Kadonaga *et al*, 1992; Barkovich *et al*, 1994; Acosta *et al*, 2005; Kinsler *et al*, 2008) and characteristic facies (Kinsler *et al*, 2012) (Figure 1). The term CMN syndrome therefore incorporates the term Neurocutaneous Melanosis, which is one particular phenotypic subset of the extra-cutaneous associations of CMN. In post-natal life affected individuals are at increased risk of cutaneous and CNS melanoma, with the peak incidence in childhood (Krengel *et al*, 2006; Kinsler *et al*, 2017a). However, the majority of congenital cutaneous and neurological abnormalities do not lead to melanoma, even when neurological disease is symptomatic, and a new classification of congenital neurological findings has been able to identify high risk groups for all clinical outcomes including all-site melanoma (Waelchli *et al*, 2015; Kinsler *et al*, 2017a).

This syndrome is caused in 80% of cases by post-zygotic mosaicism for oncogenic mutations in codon 61 of *NRAS* (Kinsler *et al*, 2013), or in other words that a single mutated cell in the embryo leads to all the cutaneous and other congenital malformations, and predisposes to malignancy by acting as a first hit in a multi-step process. Evidence for further mutation at the *NRAS* locus leading from the congenital to the malignant state has been demonstrated in a small number of cases, with loss of heterozygosity of the normal allele (Kinsler *et al*, 2013) or amplification of the mutant allele both described (Salgado *et al*, 2015). Copy-number abnormalities are also a very consistent finding in melanoma in this condition, and the specific number/pattern of multiple large gains and losses helps distinguish malignant from benign proliferations within the skin (Bastian *et al*, 2003), and also the CNS (Kinsler *et al*, 2017b).

Currently, the outlook for primary CNS melanoma in children is extremely bleak, with a recent review of our prospective in-house cohort (including these 4 cases) demonstrating 100% mortality within 9 months of diagnosis (Kinsler *et al*, 2017a), comparable to the data from a retrospective literature review (Neuhold *et al*, 2015). Our experience of systemic therapies for this disease include Dacarbazine and Temozolomide, a single experience with anti-CTLA-4 antibody therapy (Ipilimumab), and use of the mTOR inhibitor Sirolimus in three patients, none of which have had any measurable effect on symptoms or disease progression (unpublished data and Kinsler *et al*, 2017a). Due to driver *NRAS* mutations the use of selective BRAF inhibitors is contraindicated, whereas targeting the more distal MAPK pathway is a possible option. Data from a mouse model of *NRAS* codon 61 mosaicism have demonstrated inhibition of leptomeningeal disease with administration of a MEK inhibitor (Pawlikowski *et al*, 2015), with supporting evidence of the critical role of NRAS from a further murine model (Pedersen *et al*, 2013). One previous report of use in a single paediatric case was of limited use as the drug was started only days before death, although functional work *in vitro* was again supportive (Kusters-Vandevelde *et al*, 2014). These data encouraged us to test Trametinib in a small series of patients with this rare malignancy.

## Materials and methods

We therefore obtained access to the MEK inhibitor Trametinib for four children on a compassionate basis from Glaxo SmithKline (Middlesex, UK) (patient phenotypic details in Table 1). Trametinib was used initially at a dose of 0.0125 mg kg^−1^ per day in two patients (patients 1 and 2, Table 1); however, although both had symptomatic benefit (reversal of neurological signs) this was of short duration prior to rapid disease progression. When a higher dose of 0.025 mg kg^−1^ per day was established to be safe in on-going phase I paediatric studies (GlaxoSmithKline, 2016) we increased the dose to this level in two further patients (patients 3 and 4, Table 1). Prior to starting Trametinib all patients had echocardiogram, ECG, ophthalmological exam and blood tests for full blood count, urea and electrolytes, liver function tests, gamma GT and creatinine kinase.

## Results

In all four patients we saw an objective improvement in symptoms and signs. In patient 1 this was limited to recovery of a small amount of movement in both upper limbs, from day 21 after starting treatment, and after these had been paralysed for approximately 2 months prior. This limited improvement was sustained for 4 weeks, before rapid worsening in neurological status related to intracerebral haemorrhage into the intraparenchymal tumour mass. Trametinib was stopped after 49 days of therapy and she died 10 days later.

In patient 2, his parents reported a very rapid improvement in the child's mood, seeming much happier and less irritable within a few days of starting treatment. His seizures stopped, having been an ongoing problem for 3 months, and he was slowly weaned off his anti-seizure medication and oral steroids. In addition, he objectively regained power in his upper and lower limbs, as assessed by neurological examination. This improvement was sustained for 7 weeks before he suddenly decompensated, presenting with status epilepticus, and evidence of disease progression on MRI. Trametinib was stopped on day 54 of therapy and he died 3 weeks later from cord compression.

In patient 3, the drug was started much earlier in the disease, when symptoms were intermittent acutely raised intracranial pressure without other focal neurological signs. After 56 days of therapy he had not had any further episodes of raised intracranialpressure, and a reassessment MRI of the brain and spine with contrast reported stable leptomeningeal disease with improvement in the drainage of the lateral ventricles, and a further MRI at 139 days still showed stable disease. Based on previous patient disease course these findings are very encouraging. This patient then proceeded to have an entirely symptom-free period for a total of 9 months on Trametinib during which he continued to have normal neurodevelopment. This was once again, however, followed by sudden clinical and radiological disease progression, and death from spinal cord compression three weeks after stopping Trametinib.

In patient 4, there was no skin involvement, but the leptomeningeal melanoma was indistinguishable from the other cases. This has rarely been reported in the literature, and could correspond to the same post-zygotic mutation affecting only the neurological system (Dechaphunkul *et al*, 2011; Sutton *et al*, 2011; Marana Perez *et al*, 2016; Szathmari *et al*, 2016). In this patient there was marked reversal of pre-terminal cord compression one week after the increase in dose of Trametinib, and a day after starting Dexamethasone; however, the latter has not previously had a sustained effect in our patients. The patient had been admitted to a hospice in a moribund state but became suddenly asymptomatic and was discharged home, and was then symptom-free for two months before rapid disease progression, and death within a week of stopping Trametinib.

Importantly, thus far, side effects have been very few and not clinically relevant (Table 1) in any patient.

## Discussion

Trametinib (MEK inhibition) is the first medical therapy to have had any observable effect in primary *NRAS*-driven CNS melanoma in children in our cohort. Furthermore, it appears to have markedly improved disease symptoms in some cases compared to the usual course we have seen in our cohort, allowing improved quality of life for affected children in their own homes, with no clinically-significant side effects in these first four cases. The failure to stop disease progression altogether is likely to be due to the additional mutations seen in the melanoma over and above the *NRAS* driver mutation. Although the disease has eventually progressed through treatment this is an important new first step in finding therapy for this condition.

## Figures and Tables

**Figure 1:**
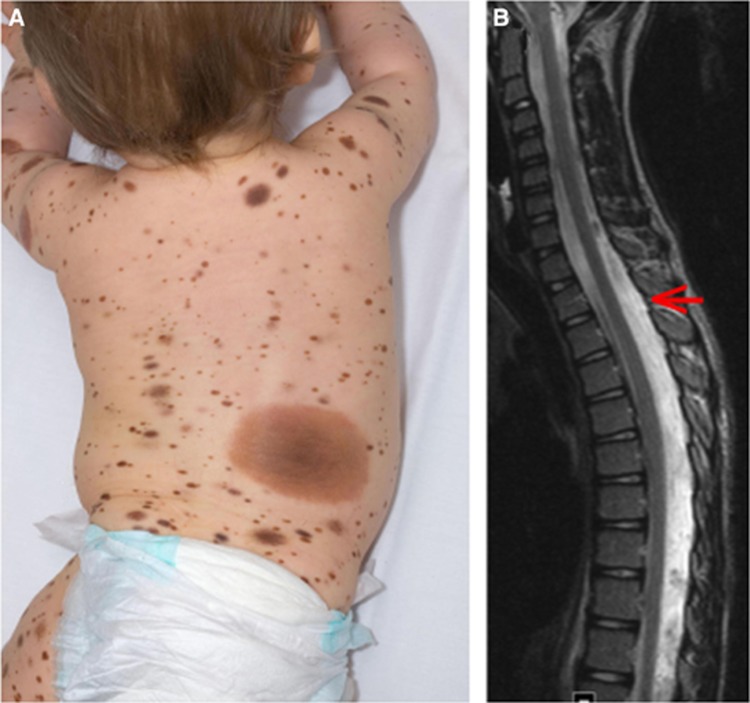
**Examples of phenotype in congenital melanocytic naevus syndrome.**Written consent was obtained for clinical photo publication. (**A**) Example of severe cutaneous phenotype. (**B**) Diffuse melanotic leptomeningeal disease—this requires biopsy and both histological and genetic testing before malignancy can be established, and should be viewed in the context of the baseline screening MRI of the CNS under 1 year. This case was leptomeningeal melanoma.

**Table 1:** Phenotype, genotype, response and outcome of patients with primary CNS melanoma treated with Trametinib, in order of commencing treatment

**Patient number**	**Sex**	**Age at diagnosis of melanoma**	**Outcome**	**Skin disease classification (Krengel *et al*, 2013)**	**Screening MRI CNS under 1 year**	**Primary melanoma site**	**Leptomeningeal tissue** ***NRAS*** **genotype**	**Initial and final Trametinib doses**	**Temporary response**	**Side effects requiring treatment**
1	Female	1.8y	Death age 2.2y	Multiple CMN, no clearly larger naevus, total >400 naevi all over body and head, no nodules Concensus classification: S3, C1, R0, N0, H1	Complex congenital neurological disease	CNS, diffuse leptomeningeal melanoma, infiltration of the underlying parenchyma, VP shunt required	c.181C>A; p.Q61K	0.0125 mg kg^−1^ per day	Limited improvement in upper limb movement	None. Temporary grade 1 rise in creatine kinase
2	Male	4.0y	Death, age 4.6y	Multiple CMN, largest in cape distribution, non-circumferential, projected adult size 20–40 cm, total 100–200 naevi, Concensus classification L1, S3, Trunk, C0, R0, N0, H1	Complex congenital neurological disease	CNS, diffuse leptomeningeal melanoma, VP shunt required	c.181C>A; p.Q61K	0.0125 mg kg^−1^ per day	Decreased irritability, cessation of seizures, improvement in limb function	None
3	Male	1.5y	Death age 2.3y	Multiple CMN, largest circumferential bathing trunk distribution, projected adult size >60 cm, total 50–100 naevi, Concensus classification: G2, S3, Trunk, C0, R1, N0, H2	Complex congenital neurological disease	CNS, diffuse leptomeningeal melanoma, VP required.	c.181C>A; p.Q61K	0.0125–0.025 mg kg^−1^ per day	Cessation of symptoms of raised intracranial pressure, stabilisation of MRI findings	None. Temporary grade 1 increase in creatine kinase
4	Female	0.9y	Death age 1.5y	No skin lesions	Not done	CNS, diffuse leptomeningeal melanoma, infiltration of underlying parenchyma, VP shunt required	c.181C>A; p.Q61K	0.0125–0.025 mg kg^−1^ per day	Cessation of symptoms of spinal cord compression and raised intracranial pressure	None

Abbreviations: CMN=congenital melanocytic naevus; CNS=central nervous system; y=years.
